# View of physicians on and barriers to patient enrollment in a multicenter clinical trial: experience in a Japanese rural area

**DOI:** 10.1186/1755-7682-3-7

**Published:** 2010-06-04

**Authors:** Hiroaki Yanagawa, Masatoshi Kishuku, Masashi Akaike, Hiroyuki Azuma, Minoru Irahara

**Affiliations:** 1 Clinical Trial Center for Developmental Therapeutics, Tokushima University Hospital, Tokushima, Japan; 2 Department of Cardiovascular Medicine, Tokushima University Hospital, Tokushima, Japan

## Abstract

**Background:**

Clinical trials in the general practice setting are important for providing evidence on the effectiveness and safety of different agents under various conditions. In conducting these trials, the participation of physicians and patient recruitment are important issues. Various investigations in the literature have reported views and attitudes of physicians on various types of clinical trials. Nevertheless, there is still little information concerning physicians participating in a clinical trial and among them, those who could not recruit any patients (unsuccessful physician recruiters).

**Methods:**

In 2003, we collaborated in a large-scale multicenter study of Japanese hypertensive patients (COPE Trial). In Tokushima University Hospital and 18 other medical institutions, we investigated the views and attitudes of unsuccessful physician recruiters in comparison with successful physician recruiters, using a questionnaire.

**Results:**

The questionnaire was provided by mail to 47 physicians and 27 (57%) responded. The response rate was 79% for successful physician recruiters compared to 43% (P = 0.014) for unsuccessful physician recruiters. More successful physician recruiters (73%) than unsuccessful physician recruiters (42%) stated they had participated and enrolled patients in previous multicenter clinical trials. A significantly higher number of successful physician recruiters than unsuccessful physician recruiters (42%; P = 0.040) considered the presence of a support system with clinical research coordinators (CRC) as the reason for participation (80%). A large number of unsuccessful physician recruiters experienced difficulty in obtaining informed consent (67%), whereas a significantly smaller number of successful physician recruiters experienced such difficulty (20%; P = 0.014). The difficulties experienced by unsuccessful physician recruiters in the trial were as follows: inability to find possible participants (100%), difficulty in obtaining informed consent (58%), cumbersome procedures (58%), difficulty in long-term follow up (33%), and insufficient tools for explanation and obtaining informed consent (8%).

**Conclusion:**

This survey showed that successful physician recruiters consider a support system with CRC of value, and that they are skillful in obtaining informed consent. These views and attitudes may have originated from past experience involving clinical trials. In this regard, we need to develop an infrastructure to enlighten physicians on this support system for the promotion of clinical trials.

## Background

An increasing number of clinical trials are seen in the general practice setting, aiming at providing evidence for the effectiveness and safety of different agents under various conditions. In conducting these trials, the participation of primary healthcare physicians in the community (general practitioners; GPs) and patient recruitment are important issues that need to be properly addressed. Various studies in the literature have reported factors affecting physicians' participation [1,2] and patient recruitment [3,4] in clinical trials, and infrastructure for clinical trials may affect these important issues.

As for the Japanese infrastructure for clinical trials, the concept of clinical research coordinators (CRC) has been introduced in clinical trials leading to drug approval (registration trials), since the introduction of the Good Clinical Practice standard approved by the International Conference on Harmonisation of Technical Requirements for Registration of Pharmaceuticals for Human Use in 1997.On the other hand, the infrastructure for other types of clinical trial remains unsatisfactory, mainly because of financial reasons, and investigators still have to do virtually everything from patient care to administrative work during the course of the study.

In 2003, we collaborated in a large-scale investigator-initiated multicenter study of Japanese hypertensive patients for the trial of a combination of antihypertensive agents (COPE Trial) [5]. This study was not a registration trial and it should be emphasized that CRC, by virtue of their contract with the Coordinating Center of the trial, supported investigators at various points, such as in recruitment and follow up of participants. Here, we present the results of investigations involving physicians who participated in the above-mentioned clinical study, with emphasis on characteristics of physicians who participated and could not recruit patients, and discuss regarding the establishment of an infrastructure for assisting physicians during participation in clinical trials.

## Methods

The collaborating study was a large-scale investigator-initiated multicenter study of Japanese hypertensive patients for the trial of a combination of antihypertensive agents (COPE Trial) [5]. The trial was conducted with the cooperation of more than 100 centers and clinics in Japan and involved 3,000 patients followed up for 3 years. The inclusion criteria was as follows: 1. Outpatients who are required a combination therapy with sitting systolic blood pressure ≥ 140 mmHg or diastolic blood pressure ≥ 90 mmHg; 2. Outpatients aged over 40 years and less than 85 years (inclusive), regardless of sex; 3. Previously untreated patients or patients who are on other therapy, which can be converted to 4 mg of benidipine; 4. Patients who can be treated with benidipine, angiotensin receptor blockers, β-blockers, and thiazide diuretics. Tokushima University Hospital (17 physicians) and 18 medical institutions (17 community-affiliated physicians from 6 hospitals and 13 physicians from 12 clinics) in Tokushima Prefecture, Japan, participated in this trial. Eligible patients were enrolled from May 2003 until November 2006, and 19 (40%) physicians eventually recruited patients in the clinical trial.

A questionnaire designed specifically for this study was composed of practical questions such as those regarding experience in trial participation and patient enrollment in the previous studies (Questions 1&2). This was followed by questions where attitudes and views were examined on a three-point scale (agree, neutral and disagree). The questions were regarding reasons for participating as investigators in the recent clinical trial (Question 3), views of physicians on the recent clinical trial after participation (Question 4), difficulties experienced by recruiting-incapable physicians in patient enrollment in the recent clinical trial (Question 5) and expectation regarding the support system provided by CRC and satisfaction from CRC participating in the recent clinical trial (Question 6). The questionnaire was provided by mail to all physicians participating in the clinical trial irrespective of patient recruitment in May 2007 after the end of the patient recruitment period, and was recovered separately from Tokushima University Hospital and other medical institutions in order to identify the category of the medical institutions to which the respondents belong.

We compared the views of physicians based on two categories: physicians who actually recruited patients in the clinical trial (successful physician recruiters) and those who could not (unsuccessful physician recruiters). Categorical variables were analyzed using the χ^2 ^or Fisher's exact test. *P *values < 0.05 were considered significant. All *P *values were based on two-sided tests.

## Results

### 1. Respondent characteristics

There were 27 respondents out of the 47 physicians (57%) included in this study. Among these respondents were 9 physicians out of 17 (53%) from Tokushima University Hospital, and 18 physicians out of 30 (60%; P > 0.05) from other medical institutions. The response rate was 79% (15 out of 19) in physicians who actually recruited patients (successful physician recruiters) compared to 43% (12 out of 28; P = 0.014) in physicians unable to recruit patients (unsuccessful physician recruiters) in the multicenter trial of antihypertensive drugs.

### 2. Physicians' past experience of participation and patient enrollment in multicenter clinical trials

As for the relationship between past experience of participation and patient enrollment in multicenter clinical trials, and patient enrollment in the recent clinical trial, there were more successful physician recruiters (73%) than unsuccessful physician recruiters (42%) who indicated having participated and enrolled patients in previous multicenter clinical trials (Table 1); however, the difference in the number of doctors was not significant.

**Table 1:** Physicians' past experience of participation and patient enrollment in multicenter clinical trials, depending on capability of recruitment in the recent clinical trial

Past experience	Successful physician recruiters (n = 15)	Unsuccessful physician recruiters (n = 12)
Participation (+)		
Enrollment (+)	11 (73%)	5 (42%)
Enrollment (-)	0	2 (16%)
Participation (-)	4 (27%)	5 (42%)

Participation (+) indicates those who have past experience of participation in multicenter clinical trials.

Participation (-) indicates those who have no past experience of participation in multicenter clinical trials.

Enrollment (+) indicates those who have past experience of patient enrollment in multicenter clinical trials.

Enrollment (-) indicates those who have no past experience of patient enrollment in multicenter clinical trials.

From the viewpoint of institutions, 45% of the physicians from Tokushima University Hospital and 28% of the physicians from other medical institutions stated that they had participated and enrolled patients in previous multicenter clinical trials (data not shown).

### 3. Reasons for participating as investigators in the recent clinical trial

We next examined and analyzed the reasons of physicians in agreeing to participate in the recent clinical trial. Many successful physician recruiters considered the presence of a support system with CRC as the reason for participation (80%), whereas a significantly smaller number of unsuccessful physician recruiters considered such reason (42%; P = 0.040) (Table 2). In terms of other reasons for participation, no significant difference was found between successful physician recruiters and unsuccessful physician recruiters.

**Table 2:** Reasons for participating as investigators in the recent clinical trial, depending on capability of recruitment

Reasons for participating as investigators	Successful physician recruiters (n = 15)			Unsuccessful physician recruiters (n = 12)		
	
	Agree	Neutral	Disagree	Agree	Neutral	Disagree
Agreement with the study aims	12 (80%)	3 (20%)	0	8 (67%)	3 (25%)	1 (8%)
Simplicity of protocol	7 (47%)	6 (40%)	2 (13%)	5 (42%)	5 (42%)	2 (16%)
Interest in agents compared in the study	6 (40%)	7 (47%)	2 (13%)	4 (33%)	5 (42%)	3 (25%)
Expectation of high probability of patient enrollment	5 (33%)	8 (54%)	2 (13%)	1 (8%)	7 (59%)	4 (33%)
Presence of support system	12 (80%)*	2 (13%)	1 (7%)	5 (42%)	3 (25%)	4 (33%)
Decision of medical institution to which physicians belong	8 (53%)	1 87%)	6 (40%)	4 (34%)	6 (50%)	2 (16%)

*: Significantly different (P = 0.040) from the value for unsuccessful physician recruiters

A smaller number of physicians from Tokushima University Hospital than from other medical institutions (33% vs 94%; P = 0.001) considered agreement with the aims of the study as the main reason for participating. Instead, the main reason of physicians from Tokushima University Hospital participating in the study was the decision by the hospital to take part in the study (66%). No significant difference in other views was observed between the institutions (data not shown).

### 4. Views of physicians on the recent clinical trial after participation

The real views of physicians on the recent clinical trial were also evaluated. Many unsuccessful physician recruiters experienced difficulty in obtaining informed consent (67%), whereas a significantly smaller number of successful physician recruiters did (20%; P = 0.014) (Table 3). No significant difference in other views was observed between successful physician recruiters and unsuccessful physician recruiters.

**Table 3:** View of physicians on the recent clinical trial after participation, depending on capability of recruitment

View of physicians on the recent clinical trial after participation	Successful physician recruiters (n = 15)			Unsuccessful physician recruiters (n = 12)		
	
	Agree	Neutral	Disagree	Agree	Neutral	Disagree
Inability to find possible participants	10 (66%)	4 (27%)	1 (7%)	10 (83%)	2 (17%)	0
Difficulty in obtaining informed consent	3 (20%)	7 (47%)	5 (33%)	8 (67%)*	3 (25%)	1 (8%)
Insufficient tools for explanation and so on	0	6 (40%)	9 (60%)	1 (8%)	5 (42%)	6 (50%)
Cumbersome procedures	7 (47%)	5 (33%)	3 (20%)	6 (50%)	4 (33%)	2 (17%)
Improvement needed in registration	6 (40%)	7 (47%)	2 (13%)	4 (34%)	4 (33%)	4 (33%)
Difficulty in long-term follow up	1 (7%)	5 (33%)	9 (60%)	4 (33%)	5 (42%)	3 (25%)
Improvement needed in follow-up system	1 (7%)	9 (60%)	5 (33%)	1 (8%)	5 (42%)	6 (50%)
Appropriate consultation system	6 (40%)	8 (53%)	1 (7%)	3 (25%)	4 (33%)	5 (42%)
Appropriate financial benefits	5 (33%)	9 (60%)	1 (7%)	4 (33%)	5 (42%)	3 (25%)

*: Significantly different (P = 0.014) from the value for successful physician recruiters

The main view of the participating physicians from Tokushima University Hospital regarding the trial was that it involved cumbersome procedures (66%), while that of the physicians from other medical institutions was their inability to find possible participants (83%). No significant difference in views was observed between institutions (data not shown).

### 5. Difficulties experienced by unsuccessful physician recruiters in patient enrollment in the recent clinical trial

Selected answers to the questions are shown in Table 4. The difficulties experienced by physicians in patient enrollment were as follows: inability to find possible participants (100%), difficulty in obtaining informed consent (58%), cumbersome procedures (58%), difficulty in long-term follow up (33%), and insufficient tools for the proper explanation of the trial and for obtaining informed consent (8%). No physician considered the following points as difficulties: negative view on the clinical trial itself, anxiety in the consultation system when some questions arise, or unsatisfactory financial benefits.

**Table 4:** Areas of difficulty experienced by unsuccessful physician recruiters in terms of patient enrollment in the recent clinical trial

Areas of difficulty	Unsuccessful physician recruiters (n = 12)		
	Agree	Neutral	Disagree
Negative view on the clinical trial itself	0	4 (33%)	8 (67%)
Inability to find possible participants	12 (100%)	0	0
Difficulty in obtaining informed consent	7 (58%)	3 (25%)	2 (17%)
Insufficient tools for explanation and so on	1 (8%)	6 (50%)	5 (42%)
Cumbersome procedures	7 (58%)	4 (33%)	1 (8%)
Difficulty in long-term follow up	4 (33%)	6 (50%)	2 (17%)
Anxiety in consultation system when some questions arise	0	5 (42%)	7 (58%)
Unsatisfactory financial benefits	0	4 (33%)	8 (67%)

### 6. Expectation regarding the support system provided by CRC and satisfaction from CRC participating in the recent clinical trial

Regarding the need for a support system as provided by CRC, 100% and 75% of the successful physician recruiters and unsuccessful physician recruiters, respectively, considered the support given by CRC as necessary for them to participate in clinical trials as their actual view. Among the 15 successful physician recruiters, 13 (87%) expressed satisfaction with the support provided by CRC in the recent clinical trial (data not shown).

## Discussion

Many clinical trials in the general practice setting have been conducted in efforts to provide evidence for the effectiveness and safety of different agents in patients with common diseases under various conditions. Most patients with common diseases are managed by not only hospital specialists, but also GPs. The participation of GPs and patient recruitment are, therefore, important issues to be properly addressed when carrying out clinical trials. Once GPs agree to participate in a clinical trial, establishing a good relationship with them and the facilitation staff is crucial to the success of the trial. In the clinical trial described here, the Clinical Trial Center for Developmental Therapeutics of Tokushima University Hospital played a key role in facilitating communication with physicians from the participating medical institutions as well as in promoting patient enrollment in the regional area. Such facilitation included regular communication by sending letters, holding seminars to gain knowledge from specialists, and providing tools for various settings, such as those for obtaining informed consent. Because of this facilitation, 19 (40%) physicians from 13 (68%) medical institutions were able to recruit patients in the recent clinical trial; however, the rest were unable to accomplish patient enrollment. In this regard, the objective of our study was to analyze the views and attitudes of physicians unable to recruit patients while participating in the trial, and to provide information useful for developing an effective strategy for improving such inability to recruit patients through the provision of support staff, including CRC, in future clinical studies.

In the present investigation, a significantly higher number of successful physician recruiters than unsuccessful physician recruiters considered the support system with CRC as the reason for their participation in the trial (Table 2). Moreover, regarding the need for support provided by CRC, 100% and 75% of the recruiting-capable and recruiting-incapable physicians, respectively, considered such support necessary for them to participate in clinical trials. In the present study, the influence of the experience of participating and enrolling patients in previous multicenter clinical trials on the capability of patient recruitment was not significant, at least partly because of the potential lack of power to detect differences. Nevertheless, successful physician recruiters may have more past experience in participating and enrolling patients in previous clinical trials, and they are more aware than unsuccessful physician recruiters of the workload of clinical trials, as well as the contributions of CRC to lessen workload and to ensure the quality of the trials [6]. The possible contribution of the CRC to recruitment outcome should be examined precisely in future studies.

A systematic review by Ross et al. [7] of 78 papers reporting barriers to the recruitment of clinicians to randomized controlled trials revealed that clinician barriers included the following: time constraints, lack of staff and training, anxieties about the impact on the doctor-patient relationship, concern for patients, loss of professional autonomy, difficulty with the consent procedure, lack of rewards and recognition, and insufficient interesting questions. In their review, the term "clinician" included all types of clinical staff, and the differing levels of participation--agreeing to participate when invited, recruiting eligible patients, and following the trial protocol--were considered together. On the other hand, only difficulties in patient enrollment were considered in the present study. After participation, a larger number of unsuccessful physician recruiters than successful physician recruiters (20%; P = 0.014) experienced difficulty in obtaining informed consent (67%) (Table 3); inability to find possible participants, difficulty in obtaining informed consent, cumbersome procedures, and difficulty in long-term follow up were pointed out as the main areas of difficulty for unsuccessful physician recruiters in terms of patient enrollment in the recent clinical trial. Since less than half of the respondents had past experience in patient enrollment, unsuccessful physician recruiters were ill-prepared for practical procedures, such as participant estimation and obtaining informed consent in the clinical trial. Difficulty in obtaining informed consent was also mentioned by Taylor et al. [3] as one of the reasons why surgical principal investigators do not enter all eligible patients in a large multicenter trial in surgical procedures against breast cancer.

It has been reported that clinicians participating in clinical trials have low recognition concerning their role in research due to inadequate research experience or training [8,9]. Japanese doctors in general still have little undergraduate or postgraduate opportunities for training in different the methodologies or regulatory issues of clinical trials. In 2004, to construct an infrastructure for implementing a clinical trial in a regional area of Tokushima, Japan, Tokushima University Hospital organized the Tokushima Network for Clinical Trials (TNCT), which is composed of regional medical institutions, in collaboration with the Tokushima Medical Association. Several seminars with clinical trial specialists as guest speakers have been given as TNCT seminars annually since 2004. In addition, monthly training seminars conducted by the Clinical Trial Center for Developmental Therapeutics of Tokushima University Hospital, which had been provided for doctors in Tokushima University Hospital from 2001, were made open to TNCT members in 2007.

As for the support system for clinical trials, in a mail survey of 221 oncologists, Somkin et al. [10] reported that the best combination of factors independently predicting enrollment related to organizational support for trials are the subspecialty of the oncologist and limitations of trial eligibility requirements, and that to increase trial participation, there is a critical need for an infrastructure to support trials, particularly additional support staff and research nurses. Since resources for clinical trials are limited, investigators who will conduct clinical trials must be aware of research infrastructure costs in future studies.

The present study is descriptive, with the potential of partly or merely justifying the inability of physicians to enroll patients in a clinical trial rather than explaining its causes. In addition, there was a significantly lower response rate among unsuccessful physician recruiters which may lead to significant bias. Nevertheless, the present study suggests that further clarification of the impact of CRC on clinical trials is necessary and future works including seminars should focus on the areas revealed by this study to be difficult for physicians in terms of patient recruitment in clinical trials. Because of the various study limitations, further study is warranted to determine the generalizability of the present findings to other Japanese areas and to international settings.

## Competing interests

The authors declare that they have no competing interests in relation to this article.

## Authors' contributions

HY conceived of the study, collected data and analyzed them, and drafted the manuscript. MK, MA, and HA participated in the design of the study and contributed to the data analysis. MI participated in the design of the study and helped to draft the manuscript. All authors read and approved the final manuscript.
